# Greetings from the new regime

**DOI:** 10.1186/s41021-018-0100-9

**Published:** 2018-06-01

**Authors:** Masami Yamada

**Affiliations:** Department of Applied Chemistry, National Defense Academy of Japan, Yokosuka City, Kanagawa, 239-8686 Japan

**Keywords:** The Japanese Environmental Mutagen Society, Genes and Environment, Asia

*Genes and Environment* (G & E) is an open access (OA), peer-reviewed journal that publishes articles across a broad range of topics. It is an official journal of The Japanese Environmental Mutagen Society (JEMS), whose mission is to discover how environmental mutagens affect all organisms (including humans) as well as the environment itself, and to apply this knowledge to the protection of human health and the environment. In April 2018, the editorial department of G & E was renewed. The new executive department consists of the former EIC, Dr. Takashi Yagi; the Editorial Director for JEMS, Dr. Takehiko Nohmi; and a new Editor-in-Chief, Masami Yamada. Together, we aim for greater heights in this important field of research.

## History of this journal

G & E has undergone transformation in a way that is consistent with the times. Environmental Mutagen Research, an academic journal of the Japanese Environmental Mutagen Society, began in 1979 and was the predecessor of G & E [1]. Initially, all JEMS publications were in Japanese, and included reviews on environmental mutagens, genotoxicity test data, and articles describing the various tests that form the basis of environmental mutagen research. The journal contributed substantially to the establishment and development of this field of research in Japan [2]. A collection of abstracts from the JEMS annual meeting was also included every year. Since then, publications on environmental mutagen research in JEMS have gradually broadened to include research on the mechanisms of mutagenesis. Several such studies in Japan have attracted the attention of not only Japanese researchers but also the rest of the world. Unfortunately, such research results tended to be published in English journals such as Mutation Research, Mutagenesis, or Environmental and Molecular Mutagenesis. Although it is convenient to have a “Japanese” tool to facilitate in-depth understanding of the research, there has been increasing movement toward disseminating Japanese research results to the rest of the world. This could be achieved through the publication of a Japanese journal in English [3]. After extensive discussion, JEMS decided to publish the journal entirely in English as “*Genes and Environment*” in 2006, which was when Dr. Minako Nagao was the EIC. Publications in Japanese were taken over by the quarterly magazine, “Jems News”. During this time, significant changes were happening in scientific publishing due to the spread of the Internet and the departure of academic journals from paper media. The next major development was G & E becoming an OA journal in 2015. There were several pros and cons to this move, but the policy of MEXT (Ministry of Education, Culture, Sports, Science and Technology) is that information should be provided to citizens for free. I think that this was the most important decision undertaken by Dr. Takashi Yagi during his term as EIC [4]. G & E was then listed in PubMed in the following year. Although the papers posted to PubMed did not initially appear as hits in online searches, they will now appear on the web.

## Features of this journal

As the name *Genes and Environment* indicates, this journal deals with “genes” and “environment.” What is the definition of genes and environment? I believe that the environment includes microscopic environments within cells as well as the larger external living environment, and thus incorporates the global or space environment as macroscopic perspectives. As genes can control certain environments, they are related to various things in the environment. The environment encompasses many things, and even the field of genetic research ranges from individual functions to massive amounts of metadata. Thus, we now need the help of researchers from different fields, including those fields that have nothing to do with genes. Meanwhile, the issue of environmental pollution, which underpinned the origin of JEMS, has not yet become outdated. It remains a hot and serious topic in BRICS countries as well as Asia [5]. From now on, I would like to actively collect manuscripts submitted from such countries. Since G & E is an official journal of JEMS, it contains features of JEMS. In other words, there are many articles relating to the regulatory sciences as well as basic research. Making full use of this feature, we will extend the journal’s impact. We will continue to pay attention to Asia where basic research output is increasing, and we will also welcome papers on the regulatory sciences, a thriving research area in Europe and the United States [6].

## The aim of this journal

We want to make G & E an attractive journal. This is easy to say; however, I think there are various avenues we can pursue to make it more attractive. First, it is probably important to have interesting articles in it. We therefore need more submissions from researchers with interesting results. The criteria authors use for deciding which journal to submit to is not only the journal’s scope but also the grade of the journal, so scientists search for the grade of journal that matches the quality of their manuscript. How is the grade of a journal measured? Most people would probably answer that is the impact factor (IF). IF is a calculation of how often articles published in the 1st and 2nd year were cited in the 3rd year. We must remember that IF is a general evaluation method, and not all papers in the same high IF journal are equally wonderful. In other words, IF is not everything. The important thing is the quality of each article. Therefore, I will make a concerted effort on the editing side to raise the quality of articles published in G & E. First, we will gather more papers by widening our readership. For this we will target Asian countries as well as the field of regulatory sciences as mentioned above. For those of you with experience of other journals, manuscripts submitted from Asian countries are not always very refined. It is easy to reject a scientific manuscript that is not in the correct format, but then the authors will continue to repeat the same mistakes. If reviewers carefully read manuscripts and provide precise commentary, then the manuscript, even if rejected, will still provide constructive input to the authors through the comments. The quality of successive manuscript submissions would therefore increase. Perseverance and effort are necessary, but I believe that the driving force in disseminating research on genes and environment in Asia is that quality research has already accumulated in this region and is waiting to be published. *Genes and Environment*, which is in the midst of becoming a fully-fledged global science journal, aims to disseminate truly influential papers to the world.
